# The Voluntary Hospitals and an Amending Act

**Published:** 1912-02-10

**Authors:** 


### The Hospital

The Workers' Newspaper of Administrative Medicine and Institutional Life, Administration, National Insurance and Health.

No. 1335, Vol. LI. SATURDAY, FEBRUARY 10th, 1912. PRICE ONE PENNY.

### THE VOLUNTARY HOSPITALS AND AN AMENDING ACT.

We are glad to note that the meeting of the chairmen of the London hospitals which have medical schools, held on February 1, whilst resulting in a decision to ascertain clearly the actual effects of the working of the Insurance Act upon the voluntary hospitals, resolved to appoint a committee consisting of the secretaries of St. Bartholomew's, St. Thomas's, Guy's, the London, Middlesex, and Dreadnought Seamen's Hospitals to collect information during the first year's working of the Act, and to report to a further meeting of the chairmen of the hospitals with medical schools. This recommendation has first to receive the approval of the Committees of Management of the hospitals immediately concerned, and they may or may not decide to make some modifications in the present methods of admission of in- and out-patients, and in the supply of drugs and dressings. We have often pointed out that the position of the London hospitals, and especially of those London hospitals which have medical schools, is much stronger in the financial sense than that of the voluntary hospitals in London without schools, and especially of the voluntary hospitals of all types situated in the United Kingdom.

No doubt the committees of the thirteen London hospitals with medical schools will take into consideration the unanimous resolution passed by the Council of the British Hospitals Association. This resolution clearly lays it down that, from the date when the Insurance Act comes into force, insured persons, except for accidents or sudden emergency, shall no longer be received at the out-patient or casualty department, unless accompanied by a certificate or introduced personally by the medical practitioner who is in attendance. In those cases which are personally introduced or produce a certificate from the medical practitioner, each patient will be examined by members of the medical staff in consultation, when they will be referred back to the same medical attendant with an expression of the opinion of the hospital physician or surgeon on the case. The British Hospitals Association went further in recommending that a list of all such insured persons, and the practitioners by whom they are sent, should be forwarded to the Insurance Committees of the district periodically. The importance of these recommendations being fully considered by the committees of the thirteen London hospitals with medical schools is that the course laid down is one which, if followed, would prove invaluable to the administrators of the Insurance Act and the Government by preventing malingering of all kinds. That the decision was unanimous, and that it has commended itself to the voluntary hospital managers throughout the country, is another reason why the London hospitals with medical schools should gravely consider these proposals before arriving at a different decision as to the policy which they will pursue in regard to insured persons.

Quite apart from the considerations just mentioned, the managers of other voluntary hospitals must remember that there is never likely to be a more favourable time for impressing the Government and the Insurance Commissioners with the importance of an immediate arrangement being come to between the managers of each voluntary hospital and the Commissioners in regard to the provision of hospital benefit, where necessary, for every insured person. The hospitals, through the British Hospitals Association, have shown their earnest desire to co-operate with the Government and the Insurance Commissioners in preventing abuses to the fullest extent. That gives them a claim to full consideration at the hands of the Insurance Commissioners, earnest desire on the latter's part to find and adopt the wisest solution in regard to this question of hospital benefit. Without such an arrangement many breadwinners up and down the country may find themselves in a worse position, when seriously ill, under the Insurance Act than they were before it came into practical working.

The Government has quite enough to face, owing to the enormous difficulties which beset them on all sides, so that the opportunity now offered of settling this question of hospital benefit upon a sound and reasonable basis is one which as prudent men they will doubtless welcome cordially. It is therefore, in our view, of the very first importance that the managers of every voluntary hospital shall make their views clear to the Commissioners or to the Council of the British Hospitals Association by writing promptly to the honorary secretary, Dr. D. J. Mackintosh, M.V.O., Western Infirmary, Glasgow. There is no time to be lost. The opportunity which the proposed amending Act offers to settle on a practical basis the difficult problem which the Insurance Act presents to hospital managers all over the country should not be let slip. It may never recur, and we very much doubt if any occasion is likely to arise in the future so favourable as the present, by the enforcement of a statesmanlike policy on the part of hospital managers throughout the country, to strengthen the voluntary system and secure its extension where necessary upon lines which must prove to the benefit of all classes of the community. The Insurance Commissioners cannot fail to grasp that it is of the first importance to prevent overlapping where medical relief is concerned, and to impress upon the mind of the public that the Act in no way helps patients needing serious operation or continuous nursing where the surroundings do not permit of either being properly carried out at the patient's home. Again, it does not in any way provide help for patients needing special treatment or advice which can only be obtained at the voluntary hospitals. Directly the Act comes into force the need for all these things will arise. Hence the necessity for supplementing the present Act in regard to hospital benefit by inserting a few clauses in the amending Act which is to be submitted to Parliament during the ensuing session.

